# Profiling the absolute and relative strength of a special operations police unit

**DOI:** 10.1186/s13102-022-00502-5

**Published:** 2022-06-20

**Authors:** Kimberly A. Talaber, Robin M. Orr, Danny Maupin, Ben Schram, Ksaniel Hasanki, Adam Roberts, Jeremy Robinson

**Affiliations:** 1 Faculty of Health Sciences and Medicine, Bond University, 2 Promethean Way, Gold Coast, QLD 4226 Australia; 2 Tactical Research Unit, Bond University, Gold Coast, QLD Australia; 3 Australian Federal Police, Canberra Unit, Canberra, ACT Australia

**Keywords:** Muscular strength, Law enforcement, SWAT, Occupational fitness

## Abstract

**Background:**

Specialist police perform high-risk tasks and are required to have, and maintain, a high level of fitness. The aims of this study were to profile the strength of a specialist police unit and to investigate whether this profile remained constant over an 18-month period.

**Methods:**

Retrospective data for 47 special operations police officers (mean initial weight = 88.84 ± 8.25 kg) were provided. Officers were tested five times over 18 months for 1 repetition maximum: bench press, squat, deadlift, and pull-up. All officers continued to participate in their typical physical conditioning programs. Repeated-measures ANOVAs with Bonferroni post-hoc adjustments or Friedman tests with Wilcoxon signed-rank tests were used to compare strength values across all five time points (TPs). Alpha levels were set at 0.05.

**Results:**

All strength values increased significantly over the 18-month period. Over the five TPs, absolute squat increased the most (+ 9%: initial mean = 125.79 ± 24.53 kg), followed by absolute bench press (+ 8%: initial mean = 109.67 ± 19.80 kg), absolute deadlift (+ 7%: initial mean = 151.64 ± 26.31 kg) and absolute pull-up (+ 4%: initial mean = 121.43 ± 14.91 kg). Relatively, the highest increase was found with the squat (+ 8%: initial mean = 1.42 ± 0.25%), followed by the bench press (+ 7%: initial mean = 1.24 ± 0.20%), deadlift (+ 6%: initial mean = 1.71 ± 0.25%) then pull-up (+ 4%: initial mean = 1.37 ± 0.15%). The period between TP3 and TP4 yielded the fewest significant increases compared with other TP differences with only absolute bench press (+ 1.7%), absolute squat (+ 1.1%) and relative bench press (+ 1.6%) changing significantly (*p* < 0.05).

**Conclusions:**

Specialist police can maintain, even increase strength, while serving in specialist units if provided with a Strength and Conditioning coach and time to train. Given changes over time, constant monitoring is required and a single timepoint may not be optimal to establish normative data.

## Background

The role of general duties police officers is to protect and support their communities, preserve peace, prevent and detect crime and to uphold and administer the law fairly and efficiently, from checking a person’s *bona fides* to attending a domestic violence incidence or effecting an arrest [1, 2]. These roles have a variety of physical requirements that include crawling, running, jumping, pushing, pulling, carrying, climbing and fighting [3]. The dynamic nature of police work exposes officers to several risks during their daily occupational activities including aggressive people, those under the influence of drugs and alcohol, dealing with people who are injured, and possible assaults [4]. Managing these situations may require aggressive manual handling. Additionally, officers may need to deal with the corresponding stress and fatigue that occurs after task completion [4].

The physical demands of policing work leads to a higher risk of injury amongst officers when compared to other professions [3] with rates of formally reportable injuries of up to 2.5 per officer—per year reported [5]. To combat this risk, a high level of physical fitness is required by police officers to assist them to safely perform their duties [3]. The term ‘physical fitness’ encompasses many aspects of health, inclusive of muscular strength. Muscular strength is defined as a muscle’s ability to generate maximal force at a specific velocity [6]. In addition to numerous other benefits, including a positive sense of wellbeing, muscular strength is also associated with task performance in law enforcement populations [7–9]. Due to this relationship, several training programs are administered early in a police officer’s career whilst they are still recruits in order to condition them to meet the occupational requirements of police work [10–13]. Despite the effect of training interventions being reported in police recruits [10–13] and general police officers [8, 14], little is known about training practices in specialized police units, due to strict security and sensitivity surrounding these populations.

Specialist police units encompass a subset of officers under the broad scope of police. These officers are specially trained personnel that perform high risk tasks, like hostage negotiations, effecting a warrant, high risk security, and forced entry using specialist equipment—often while working in environments that are often violent and unpredictable [15–17]. As their role goes above and beyond that of general duty police officers, specialized weaponry and tactics are often employed [16, 17], which results in additional body armor and enhanced weapons, adding to the occupational load a specialist police officer will carry [18]. In order to function with this increased load, weighing from 18 to 23 kg on average and excluding other specialist equipment (e.g., door breaching equipment, ballistic shields) [16, 17, 19, 20], this elite group of officers require remarkable physical fitness and mental rigor, above that of general police, in order to successfully complete their missions [21]. Often these specialist police are considered to be at a higher level of fitness than the general police officer, and may be considered elite athletes [22]. However, minimal time to train, occupational taskings, sleep deprivation and fatigue may all expose specialist police officer to sub-optimal conditions to complete the required training to maintain these fitness levels.

Another consideration is the effect of occupational demand on physical performance over time in specialist police units. Most physical profiles of police officers report values measured only once or twice, whether it be a baseline measure or pre- and post-training values [8, 10, 11, 14, 23–26]. This methodology provides a single observation of the population’s physical capacity which may not yield an accurate portrayal of performance over the span of their careers. Documenting measurements in the same officers over time is necessary to track physical capacity and to evaluate potential factors influencing any fluctuations.

Given the high levels of strength required in the specialist police officer’s domain and the minimal research to date in specialized units, the aims of this study were to profile the strength, both absolute and relative, of a specialist police unit and to investigate whether this profile remained constant over an 18-month period of oriented strength and conditioning training.

## Methods

### Subjects

Retrospective data from 47 male elite police officers were provided according to strict security protocols regarding the protection of personnel identification. No demographic data (other than gender) were provided with the only anthropometric data provided being body weight (mean at commencement of study = 88.8 ± 8.25 kg). This limitation of data for security reasons in law enforcement research is not uncommon [9, 27, 28]. Ethics approval, for the collecting of non-identifiable retrospective data collected as part of training processes with a waiver for informed consent was granted through the Bond University Human Research Ethics Committee. All methods were performed in accordance with relevant guidelines.

### Procedures

Data were collected on five occasions over an 18-month period, being: TP 1 to TP 2 and TP 2 to TP 3 (3–4 months), and TP 3 to TP 4 and TP 4 to TP 5 (5–6 months). All strength testing was performed over two days at each time point. 1RM bench press and 1RM deadlift measures were performed on Day 1. 1RM squats and 1RM pull-ups were performed on Day 2. Prior to testing, participants performed a 10-min warm-up which included supine glute bridges, side-lying clams, bodyweight squats, single leg Romanian deadlifts (bodyweight), bodyweight lunges in clock pattern, alternating forward lunges with overhead reach, Hindu push-ups with rotation, clap push-ups, supine alternating leg and lumbar rotations, and 5–10 kg medicine ball slams.

#### 1RM testing

1RM is defined as the maximal force a muscle or muscle group can exert for one single voluntary effort [29]. The 1RM test is a gold standard for assessing strength in non-laboratory situations the power rack. Officers were then instructed to place their hands on the bar with a grip slightly wider than shoulder-width as [30]. The test appears to be extremely diverse and can be conducted for a range of exercises [29]. Protocols for the 1RM measurements are described below. For each of these 1RM assessments, deviations from the described lifting protocols, while not specifically recorded by the strength and conditioning coaches, were considered a failed lift. This approach was taken to ensure technical competency was maintained by officers and that reliable data was captured to allow for comparisons to future tests. Further reliability was enforced with the same coach leading each 1RM lift over the duration covered in this study. A minimum of three minutes rest between all lifts was allowed to ensure sufficient recovery prior to the next lift attempt.

### 1RM squat

A 20 kg Pendlay brand barbell, Gym Garage brand bumper weight plates and a Hammer Strength power rack was used for the back squat protocol. Under the guidance of two Strength and Conditioning (S&C) coaches, officers were instructed to position themselves under the squat bar that was secured on permitted) and ensure that the barbell rested on their upper back (below the seventh cervical spinal process and upon the trapezius muscle). Officers were instructed to maintain a stance where feet were just outside hip-width apart. Next, officers lifted the barbell from the rack supports and took two steps backward to get into position. Officers performed the squat to a depth where knees and hips were flexed to 90° (femurs parallel to floor) and returned to starting position, achieving full hip and knee extension.

### 1RM deadlift

For assessment of the deadlift, a 24 kg diamond-shaped bar was used (Australian Barbell Company) with Gym Garage brand weight plates. Officers were instructed to position themselves inside the bar with feet just outside hip-width apart. To start the lift, arms were fully extended at their sides with heels flat on the ground. The officers lifted the bar vertically until hips and knees were fully extended. Once the S&C coach instructed “down”, the officers lowered the bar in a controlled manner.

#### 1RM bench press

Using a 20 kg Pendlay bar, Gym Garage weight plates and a Hammer Strength power rack, officers were instructed to position themselves supine on a horizontal bench, with feet flat on the floor ensuring shoulder blade and buttock contact on the bench. Hand grasp was slightly wider than shoulder-width apart to allow for 90° elbow flexion at the lowest position during the press. Officers removed the weighted bar from the rack with arms fully extended at chest level. Next, they lowered the bar so that it touched their chest, then raised it back to the starting position before returning it to the rack supports. One S&C coach supervised and recorded this test.

#### 1RM pull-up

The starting position for the pull-up involved the officer’s hands being pronated with a grip wider than shoulder-width apart to ensure 90° elbow angle at the point when upper arms were parallel to the ground. Officers crossed their ankles and bent their knees to 90°. A Dan Baker Strength brand weight belt of appropriate weight was positioned such that the weight hung in front of the officer’s body. To start the pull-up, the officer’s chin had to rise above the level of the bar without any leg swinging. Then the officer lowered themselves in a controlled manner. The 1RM weight documented was the officer’s body weight plus the additional weight lifted.

All officers continued to participate in their typical physical conditioning programs which were provided by a full time S&C coach working in the unit. Sessions were typically conducted during work time. Limited information was provided regarding the specific S&C program. Generally, the S&C coach applied a block periodization model for strength training [31]. The block periodization model (see Table 1) focused on breaking down specific training periods into 2–6-week periods each encompassed one of three training different blocks (accumulation, transmutation, and realization blocks) followed by a recovery block. The coach and officers were blinded to the testing requirement (i.e., for research).

**Table 1:** Program periodisation overview

Training Block	Accumulation 2–6 weeks	Transmutation 2–6 weeks	Realization 2–6 weeks	Deload/recovery 2–4 weeks
Performance goals	General adaptation strength Endurance/Hypertrophy	Specific adaptation max strength power "Occupational Task Readiness””	Peaking max strength power conversion “Operational Deployment”	Unloading for next block
Volume	High	Medium	Low	Low
Frequency	Medium 3–4 sessions per week	Medium 3–4 sessions per week	Medium 2–3 sessions per week	Low 2–3 sessions per week
Intensity	Low 55–70% training max	Medium 70–85% training max	High 85–95% training max	Low 50% training max

### Statistical analysis

Data were imported into SPSS Version 23.0 (IBM Corporation, Armonk, NY, USA) for statistical analysis. Descriptive analysis, including means and standard deviations were calculated for bodyweight and for absolute and relative strength values at each TP. Shapiro–Wilk tests and visual inspection of frequency histograms were used to determine distribution normality. For the normally distributed data groups, repeated-measures ANOVA analyses were utilised with Bonferroni post-hoc adjustments performed to compare individual values across 18 months with statistical significance set at *p* < 0.05. For the remaining data groups with abnormal distributions, Friedman tests were used to determine if there were significant differences in scores over 18 months. Then Wilcoxon signed-rank tests were conducted to determine where the significant changes occurred (*p* < 0.05). Given the high level of fitness of specialist police [21], effect sizes (Cohen’s *d*) reported for highly trained persons (at least 5 years of training) for strength training research were used whereby a value less than 0.25 was considered a trivial effect, 0.25 to 0.50 a small effect; 0.50 to 1.0 a moderate effect; and > 1.0 and a large effect [32].

## Results

**Fig. 1:**
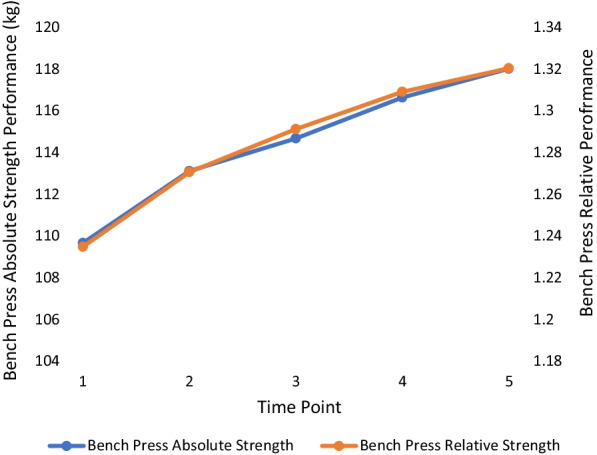
Bench press performance over time. Key: TP 1 to TP 2 and TP 2 to TP 3 = 3–4 months: TP 3 to TP 4 and TP 4 to TP 5 = 5–6 months

**Fig. 2:**
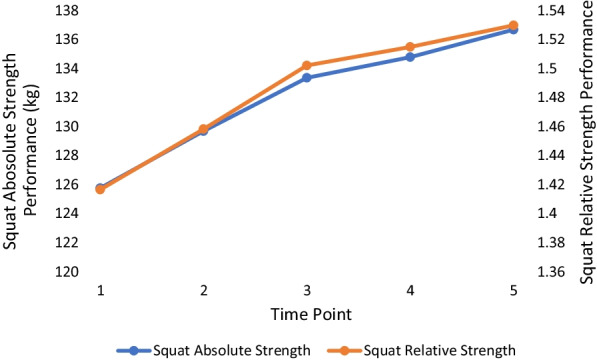
Squat performance over time. Key: TP 1 to TP 2 and TP 2 to TP 3 = 3–4 months: TP 3 to TP 4 and TP 4 to TP 5 = 5–6 months

**Fig. 3:**
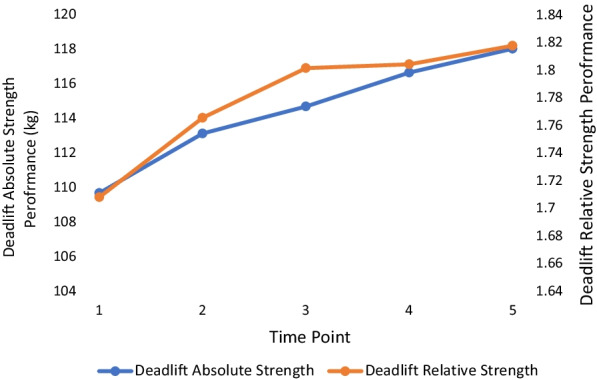
Deadlift performance over time. Key: TP 1 to TP 2 and TP 2 to TP 3 = 3–4 months: TP 3 to TP 4 and TP 4 to TP 5 = 5–6 months

**Fig. 4:**
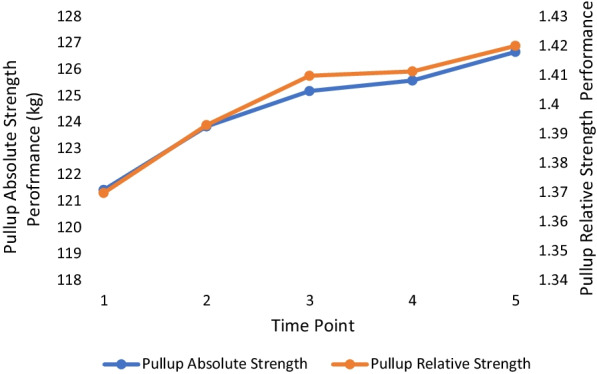
Pullup performance over time. Key: TP 1 to TP 2 and TP 2 to TP 3 = 3–4 months: TP 3 to TP 4 and TP 4 to TP 5 = 5–6 months

The means and standard deviations for all outcome measures with corresponding statistical significance are outlined in Table 2 with effect sizes in Table 3. There were no significant changes in body weight at any point over the 18 months. All absolute and relative strength measures changed significantly between TP 1 and TP 5. For each absolute and relative measure of strength, the lowest value occurred at the first TP, and the highest value occurred at the last TP, with each trending upwards over the 18 months. Over the five TPs, absolute squat increased the most (+ 9%: initial mean = 125.79 ± 24.53 kg, Cohen’s *d* = 1.39), followed by absolute bench press (+ 8%: initial mean = 109.67 ± 19.80 kg, Cohen’s *d* = 1.50), absolute deadlift (+ 7%: initial mean = 151.64 ± 26.31 kg, Cohen’s *d* = 1.22) and absolute pull-up (+ 4%: initial mean = 121.43 ± 14.91 kg, Cohen’s *d* = 0.96). A similar result was found in relative terms with the highest increase found with the squat (+ 8%: initial mean = 1.42 ± 0.25%, Cohen’s *d* = 1.13), followed by the bench press (+ 7%: initial mean = 1.24 ± 0.20%, 1.30), deadlift (+ 6%: initial mean = 1.71 ± 0.25%, Cohen’s *d* = 1.1.0) then pull-up (+ 4%: initial mean = 1.37 ± 0.15%, Cohen’s *d* = 0.98). The period between TP3 and TP4 yielded the fewest significant increases compared with other TP differences with only absolute bench press (+ 1.7%), absolute squat (+ 1.1%) and relative bench press (+ 1.6%) changing significantly (p < 0.05). Figures 1, 2, 3 and 4 show the change in mean lift performance over time.

**Table 2:** Bodyweight, strength data and overall change (Mean ± SD) for five TPs over 18 months

	TP 1	TP 2	TP 3	TP 4	TP 5	Overall ∆ (TP1 → TP5)
Bodyweight (kg)	88.84 ± 8.25	89.07 ± 8.27	89.00 ± 8.58	89.24 ± 8.78	89.52 ± 8.73	↑0.68 ± 1.70
Bench Press Absolute Strength (kg)	109.67 ± 19.80^a,b,c,d^	113.05 ± 19.67^b,c,d^	114.67 ± 20.17^c,d^	116.62 ± 19.94^d^	118.00 ± 19.01	↑8.33 ± 5.58*
Bench Press Relative Strength (ratio^$^)	1.24 ± 0.20^a,b,c,d^	1.27 ± 0.20^c,d^	1.29 ± 0.21^c,d^	1.31 ± 0.20	1.32 ± 0.19	↑0.09 ± 0.07*
Squat Absolute Strength (kg)	125.79 ± 24.53^a,b,c,d^	129.71 ± 24.32^b,c,d^	133.38 ± 24.58^c,d^	134.81 ± 25.01^d^	136.70 ± 25.08	↑10.91 ± 7.84*
Squat Relative Strength (ratio^$^)	1.42 ± 0.25^a,b,c,d^	1.46 ± 0.25^b,c,d^	1.50 ± 0.26^d^	1.51 ± 0.27^d^	1.53 ± 0.26	↑0.11 ± 0.10*
Deadlift Absolute Strength (kg)	151.64 ± 26.31^a,b,c,d^	157.09 ± 27.54^b,c,d^	159.96 ± 27.88^d^	160.83 ± 27.67^d^	162.60 ± 28.97	↑10.96 ± 8.99*
Deadlift Relative Strength (ratio^$^)	1.71 ± 0.25^a,b,c,d^	1.76 ± 0.27^b,c,d^	1.80 ± 0.28	1.80 ± 0.27	1.82 ± 0.28	↑0.11 ± 0.10*
Pull Up Absolute Strength (kg)	121.43 ± 14.91^a,b,c,d^	123.83 ± 14.81^b,d^	125.17 ± 14.93^d^	125.57 ± 14.20^d^	126.66 ± 15.59	↑5.23 ± 5.41*
Pull Up Relative Strength (ratio^$^)	1.37 ± 0.15^a,b,c,d^	1.39 ± 0.14^b,d^	1.41 ± 0.14	1.41 ± 0.13	1.42 ± 0.14	↑0.05 ± 0.05*

^$^Relative values calculated by [absolute strength value / officer bodyweight]

^a^Significant difference from TP 2 (*p* < 0.05), ^b^Significant difference from TP 3 (*p* < 0.05), ^c^Significant difference from TP 4 (*p* < 0.05), ^d^Significant difference from TP 5 (*p* < 0.05)

^*^Significant difference between TP 1 and TP 5 (*p* < 0.05)

**Table 3:** Calculated effect sizes

	TP2–TP1	TP3–TP1	TP4–TP1	TP5–TP1	TP3–TP2	TP4–TP2	TP5–TP2	TP4–TP3	TP5–TP3	TP5–TP4
BW	0.22	0.12	0.23	0.4	0.06	0.08	0.24	0.16	0.34	0.28
ABP	1.04	0.88	1.31	1.50	0.32	0.86	1.10	0.67	0.81	0.47
RBP	0.85	0.85	1.28	1.30	0.36	1.00	0.87	0.49	0.56	0.25
AS	0.93	1.16	1.23	1.39	0.64	0.79	0.94	0.37	0.76	0.59
RS	0.76	1.06	1.07	1.13	0.60	0.66	0.75	0.27	0.52	0.41
AD	0.93	0.99	1.15	1.22	0.42	0.57	0.72	0.20	0.44	0.42
RD	0.78	0.91	1.07	1.10	0.51	0.56	0.64	0.04	0.21	0.25
AP	1.11	1.10	0.90	0.96	0.42	0.42	0.56	0.13	0.37	0.30
RP	1.10	1.08	0.80	0.98	0.45	0.32	0.51	0.03	0.18	0.15

Key: BW: bodyweight, ABP: absolute bench press, RBP: relative bench press, AS: absolute squat, RS: relative squat, AD: absolute deadlift, RD: relative deadlift, AP: absolute pullup, RP: relative pullup. Interpretation: trivial = 0.25, small = 0.25–0.50, moderate = 0.50 to 1.0, large > 1.0 (32)

## Discussion

The aims of this study were to profile and monitor the strength of a specialist police unit over an 18-month period to determine the ability of this population to maintain strength. Overall, the results of this study found a significant upward trend of both absolute and relative strength values while bodyweight remained unchanged. This consistent body weight suggests that the increases in relative strength were due to increases in absolute strength rather than changes in body weight.

The 5–6-month period between TP 3 and TP 4 yielded the smallest change in all four absolute measures and three of the relative strength measures (squat, deadlift, and pull-up). Unfortunately, due to the classified nature of this population, it was difficult to determine the cause for this plateau. A potential reason could be due to the focus of their conditioning schedule at that time point (i.e. training to increase metabolic fitness rather than muscular strength) [33, 34]. Other potential reasons could be a change in typical routine (e.g. supporting an event, running recruiting courses or other general police work) [16, 35] or a change in team dynamics (recruitment of new members increasing the need for task specific training), any of which may prevent the participating officers from making significant strength gains during this period by impacting on their ability (or desire) to train. Another influence may have been location of training and equipment restrictions the officers were subjected to, based on their job requirements (e.g., a week of weapon training at a field firing range). Occupational requirements including extensive travelling and varied work environments requiring significant load carriage may lead to minimal opportunities for strength improvements [36].

The most commonly reported strength measures in law enforcement are grip strength, 1RM bench press, 2–5 RM leg press, and isometric leg and back strength [37]. Previously reported bench press results in law enforcement have ranged from 64.3 kg [38] to 124.6 kg [14] for sworn officers and 81.5 kg [39] to 113 kg [10] for male police recruits which compare well to the results presented in this study. American Special Weapons And Tactics (SWAT) members have measured a mean 1RM bench press as 106.8 kg [26], which is lower than any mean 1RM bench press value presented in this study. A potential reason for this difference is the ongoing dedicated strength and conditioning program that was provided and supervised by a qualified coach in this study. 1RM values for squat, deadlift and pull-up are far less common in current literature, making comparison of other strength measures difficult.

Historically, profiling absolute and relative strength values in law enforcement populations is done either at a single snapshot in time [14, 23, 24], or reported as pre- and post-training values [10, 11, 25]. The current breadth of literature lacks ongoing strength measures, which limits applicability and insight into how individual and team strength values may change over time. If the special operations police officers in this study were compared to another population’s strength measures, the comparative result may be different depending on the TP when the comparison occurs. For example, when comparing the results of this study to a population of police academy cadets, who demonstrate a mean 1RM bench press value of 113.00 kg [10], this study’s population demonstrated a lower mean 1RM bench press value (109.67 kg) at TP 1. However, they then demonstrated a similar mean value at TP 2 (113.05 kg), and higher mean values at TP3, TP 4 and TP 5 (114.67 kg, 116.62 and 118.00 kg respectively). Relying simply on a single snapshot in time, rather than repeated documentation at regular intervals, may not provide an accurate basis for comparison to other populations. In fact, in this study, no strength measure that was reported at TP 1 remained unchanged by TP 5. This study has demonstrated how much change can be seen in the same officers over 18 months.

There has been a recent shift in the portrayal of the physical abilities law enforcement officers (and other tactical officers) must maintain, which is that of a “tactical athlete” [22]. Supporting this term is the remarkable state of physical fitness required of tactical professionals that is not dissimilar to that of an elite athlete in terms of flexibility, muscular strength, anaerobic endurance and cardiorespiratory endurance [22]. Differences lay, however, in the allowable fluctuations within these components to perform optimally. Elite athletes of sports such as handball [40], basketball [41] and rugby union [42] have designated time off during training season so as to prevent fatigue and optimise performance for competitions. They also take time off post-season for recovery, a notion which is non-existent in special operations police units due to ongoing job demands, rather than seasonal competition.

It is worth consideration that typical strength training involves maximal effort [29] which requires long bouts of rest and a potential for delayed onset muscle soreness. Specialist law enforcement personnel may not be able to train at maximal exertion whilst on-duty as they must always be able to respond to, and perform effectively in, emergency situations when required, at short notice [22]. This may minimise the ability for strength gains due to the trade-off between training and maintaining the ability to perform life-saving, high intensity work that is physically and mentally exhausting [22]. Furthermore, unlike athletes, who perform discrete tasks which can be profiled [43], specialist police officers must be able to perform a myriad of tasks, from short duration explosive events (like a victim drag), to sustained duration events (like searching woodlands for an offender), or those that require a combination of long durations of waiting wearing full loads of 22 + kg and then performing an explosive task in life critical circumstances [16, 20]. As such, where one task could require levels of strength and power, another would be reliant on aerobic fitness [44]. Given the concerns of concurrent training (i.e. training for strength and aerobic fitness) [45], the ability to just optimise strength development presents as a challenge. Furthermore, the strength requirements of a given task may change. For example, research by Orr et al., [9] found that where absolute strength was more important than relative strength for specialist police officers conducing a victim drag with an 85 kg mannequin, relative strength was more important for their general load carriage requirements (40 kg carried load).

There are some limitations to this study which need to be acknowledged. Strength evaluations performed during this study did not occur at a consistent frequency with gaps of either 3–4 months (TP 1 to TP 2 and TP 2 to TP 3) or 5–6 months (TP 3 to TP 4 and TP 4 to TP 5) which may skew perception of strength gains (i.e., changes seen after 3 months compared with changes seen after 6 months). A second limitation is the classified nature of the training program particulars during each time period. The details of the aim and progress of the periodisation of training, or whether it was a new training program is unknown. Finally, both the small sample size and limited demographic detail could be viewed as a limitation in this study. However, the size of the sample in this study was larger than many studies involving specialist police populations [19, 20, 46].

Future research profiling absolute and relative strength values of special operations police units, as well as tracking these values over time, would benefit all stakeholders invested in tactical training programs. Additional monitoring of sleep, stress, and pain levels could provide further context into performance improvements. Despite the additional time commitment it takes to measure these outcomes regularly, gathering knowledge of strength patterns in a specialist police unit may be used to optimise that unit’s performance. Understanding how levels of strength may vary can help inform future training programs and lead to organizational discussions surrounding group tactics. This may prevent or minimize factors that could compromise the physical strength of specialist officers and hence the integrity of their unit.

## Conclusion

The results of this study suggest that strength testing over time may give a more accurate picture of strength capabilities of special operations police units. In addition, it appears that despite occupational commitments and sub-optimal conditions, special operations police officers are able to maintain high levels of absolute and relative strength, both important factors in occupational task performance and injury prevention, if provided with a Strength and Conditioning coach and time to train.

### Practical applications

Strength assessments should be performed over multiple time periods to ensure a more accurate representation of an individual’s or unit’s strength profile. Trainers can then evaluate possible influencing factors between two TPs in order to optimize physical training. Caution should be used when viewing research that reports strength demonstrated at only one time point.

## Data Availability

As the data is drawn from a tactical population, the data and materials will only be made available upon a formal specific request made to the corresponding author who will seek approval from relevant agencies. A formal request will not infer approval.

## Abbreviations

RM: Repetition maximum
S&C: Strength and conditioning
SWAT: Special weapons and tactics
TP: Time points
BW: Bodyweight
ABP: Absolute bench press
RBP: Relative bench press
AS: Absolute squat
RS: Relative squat
AD: Absolute deadlift
RD: Relative deadlift
AP: Absolute pullup
RP: Relative pullup
